# Supplementary material for: Copy number-dependent DNA methylation of the Pyricularia oryzae MAGGY retrotransposon is triggered by DNA damage
Source: Commun Biol. 2021 Mar 19;4:351. doi: 10.1038/s42003-021-01836-5 (PMC7979813; doi:10.1038/s42003-021-01836-5)
Supplement: Supplementary file 2 — Supplementary Information [file 42003_2021_1836_MOESM2_ESM.pdf]

Supplementary Table 1. Primer pairs used in qMSRE assay of MAGGY

Positions	Sequences
212S	5'-AACAACCACCTGCTACCCTGTA-3', 5'-CTCGGTCGAATCGGTTAAGAT-3'
817H	5'- CTTTCTGAACTTCCAGACC-3', 5'- CGGTCTTTGTTTGAGGTCATCT-3'
1109H	5'- GAACGTAGATTTCGCCGAGTAT-3', 5'- GTCCTGGAGCTCTTCCGATATT-3'
1684H	5'- AGCAGCCGAAGTTCGAAAAAC-3', 5'- CGGATCGTTGGTTATATTCCTC-3'
2152H	5'- TACGTTTGACAGCGACTATTGC-3', 5'- GGTCGGTCAGCCTGGTACT-3'
3023H	5'-AGAACATTTGAAACTCGTACTGGA-3', 5'-TGGTTTCCGTCACGAAGAAT-3'
3697H	5'- ACCTGGAATATTTCACGACGAC-3', 5'- CATATCCTCTGACCTCCTGGTT-3'
4148H	5'- GACCTGCTGTCCCGAGAATA-3', 5'-CATGAGCGTTCAGGAGTGG-3'
4750H	5'- GTTTTACGCGACTTACGGATTC-3', 5'- GCTTGTTCCAGAATGGCTTC-3'
5113H	5'- TGCCGTCGTCTATGAGAGTACA-3', 5'- TTCTCTCTCACCTTCCACTTCC-3'
5598S	5'-CTACGTCTTCGTCAACAACCAC-3', 5'-AACCCGGGCATATTTTTATTTT-3'

Supplementary Table 2 Mapping of MeDIP reads to different fractions of the *Pyricularia oryzae* genome

	total reads	CDS	Intron	Intergenic	undetermined	RNA genes						Repeats[4]			
						26S rRNA	18S rRNA	5.8s rRNA	5s rRNA	tRNA	snoRNA/ snRNA	non-LTR	LTR	DNA	unclassified repeats
WT-cont[1]															
No of reads	40,608,444	18,141,382	4,547,429	15,150,190	770,955	432,666	239,425	9,396	6,255	18,400	996	195,808	509,578	60,773	525191
percentage		44.67%	11.20%	37.31%	1.90%	1.07%	0.59%	0.02%	0.02%	0.05%	0.00%	0.48%	1.25%	0.15%	1.29%
WT-5mC[2]															
No of reads	7,843,032	2,276,984	622,908	3,028,452	620,006	229,787	114,758	6,400	1,254	2,140	81	194,493	544,482	40,667	160620
percentage		29.03%	7.94%	38.61%	7.91%	2.93%	1.46%	0.08%	0.02%	0.03%	0.00%	2.48%	6.94%	0.52%	2.05%
fold enrichment		0.65	0.71	1.03	4.16	2.75	2.48	3.53	1.04	0.60	0.42	5.14	5.53	3.46	1.58
DKO-5mC[3]															
No of reads	13,293,983	3,358,556	944,114	5,415,403	1,238,545	293,672	131,917	7,171	2,361	3,567	114	313,314	1,201,419	92,275	291555
percentage		25.26%	7.10%	40.74%	9.32%	2.21%	0.99%	0.05%	0.02%	0.03%	0.00%	2.36%	9.04%	0.69%	2.19%
fold enrichment		0.57	0.63	1.09	4.91	2.07	1.68	2.33	1.15	0.59	0.35	4.89	7.20	4.64	1.70

[1]WT-cont, genomic DNA reads of the wild-type strain without MeDIP

[2]WT-5mC, MeDIP reads of the wild-type strain

[3]DKO-5mC, MeDIP reads of the Δddnm1/Δrhm51 strain

[4]non-LTR, non-LTR retrotransposons; LTR, LTR-retrotransposons; DNA, DNA transposons

Supplementary Table 3 Genomic locations showing different levels of MeDIP read coverage between the wild-type and ∆ddnm1/∆rhm51 strains

Chromosome	Start position	End position	Sequence	Fold Change	P-value[1]	WT - RPKM	∆ddnm1/∆ rhm51 - RPKM
Supercontig_8.1	1196000	1197000	Vector	∞	6.24E-03	0.00	14.24
Supercontig_8.1	6672000	6673000	MAGGY	-28.2	7.21E-04	54.75	1.94
Supercontig_8.2	2439000	2440000	Intergenic	835.1	2.65E-05	0.05	41.80
Supercontig_8.2	3751000	3752000	MGG_01486	-90.3	1.76E-03	668.80	7.41
Supercontig_8.2	3752000	3753000	MGG_01486	-65.0	2.36E-03	702.55	10.80
Supercontig_8.4	1168000	1169000	Vector	-115.4	9.69E-08	2197.75	19.04
Supercontig_8.4	1169000	1170000	Vector	-174.4	3.38E-08	3352.08	19.22
Supercontig_8.4	1170000	1171000	Vector	-55.3	8.10E-07	2524.53	45.61
Supercontig_8.4	1171000	1172000	Vector	-399.7	9.60E-07	3703.83	9.27
Supercontig_8.4	1172000	1173000	Vector	-542.8	1.58E-07	1137.18	2.10
Supercontig_8.4	1974000	1975000	MAGGY	-19.9	9.68E-03	17.50	0.88
Supercontig_8.5	3686000	3687000	MGG_00208	-17.3	3.73E-03	71.59	4.13
Supercontig_8.6	3250000	3251000	MAGGY	-16.0	4.97E-03	23.58	1.47
Supercontig_8.8	467000	468000	MAGGY	-41.3	2.86E-04	321.78	7.80
Supercontig_8.8	512000	513000	Vector	-86.6	5.41E-05	269.69	3.12

[1]Significant difference (p<0.01, empirical analysis of DGE (the CLC Genomics Workbench software))

Supplementary Table 4 Fungal strains used in this study

Strain	Genotype	reference
P. oryzae Br48	wild type	Urashima et al. 1998[37]
Δddnm1	ddnm1::hygR	This study
Δrhm51	rhm51::hygR	This study
Δddnm1/Δrhm51(Δ11350)	ddnm1/rhm51::hygR	This study
Δmorad55	morad55::genR	This study
Δmorad57	morad57::genR	This study
Δddnm1/Δmorad55	ddnm1::hygR, morad55::genR	This study
Δddnm1/Δmorad57	ddnm1::hygR, morad57::genR	This study
Δrhm51/Δmorad55	rhm51::hygR, morad55::genR	This study
Δmodcl1	modcl1::hygR	Kadotani et al. 2004[20]
Δmodcl2	modcl2::hygR	Kadotani et al. 2004[20]
Δmoago1	moago1::hygR	Nguyen et al. 2018[21]
Δmoago2	moago2::hygR	Nguyen et al. 2018[21]
Δmoago3	moago3::hygR	Nguyen et al. 2018[21]

Supplementary Figure 1. DNA methylation assays of MAGGY

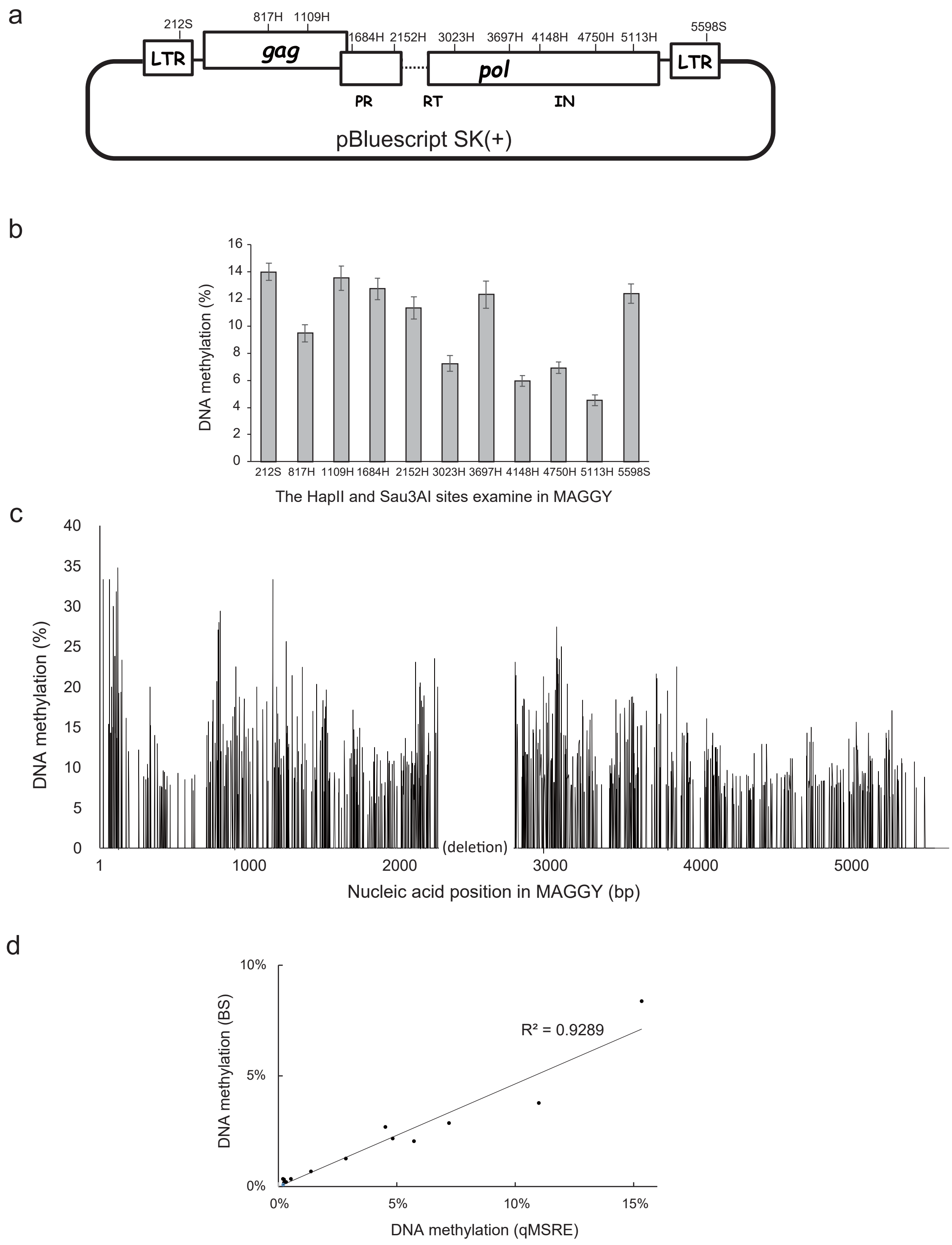

(a) A schematic representation of pMGY-ΔRT having a 513 bp deletion (shown as a stippled line) in the reverse transccriptase domain of the LTR retrotransposon MAGGY. The positions of *Hap*II (H) and *Sau*3AI (S) sites examined for DNA methylation are indicated. LTR, long terminal repeats; gag, group-specific antigen; pol, polyprotein; PR, protease; RT reverse transcriptase; IN, integrase. (b) The rates of DNA methylation at different sites in MAGGY were assessed by the methylation sensitive restriction enzyme-quantitative PCR (qMSRE) method. Eleven pairs of primers listed in Table S1 were used in the analysis. Each bar represents the average of three technical replicates (±SE). (c) The rates of DNA methylation at a total of 595 different positions in MAGGY assessed by whole genome bisulfate sequencing (Bis-seq). A *Pyricularia oryzae* transformant with approximately ten copies of pMGY-ΔRT was used in this assay. (d) Relationship between rates of DNA methylation assessed by conventional bisulfate sequencing (BS) and qMSRE methods in thirteen *P. oryzae* transformants having different copies of pMGY-ΔRT. In the BS analysis, an approximately 600bp fragment in the reverse transcriptase domain of MAGGY was amplified and cloned into pBluescript SK(+). Ten to fifteen clones were sequenced for each transformant.

Supplementary Figure 2. Construction of double deletion mutants of *Rhm51* and *MGG_15577*

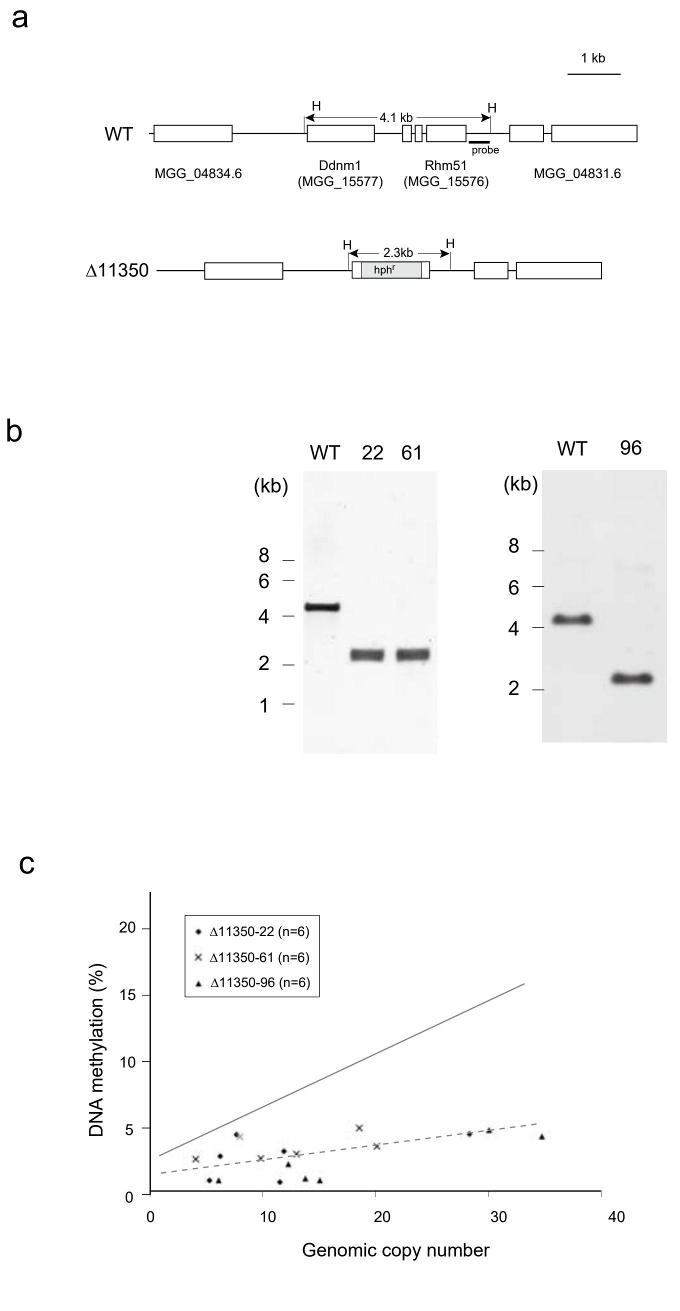

(A) Schematic representation of the *Rhm51* and *MGG_15577* loci. The open boxes indicate exons and predicted genes. The bold line shows the postion of the probe used in the Sourhtern blot analysis. The sizes of the *Hin*dIII fragments are given with arrowed lines. H, *Hin*d III. (b) Genomic Southern analysis of the double deletion mutants (22, Δ11350-22; 61, Δ11350-61; 96, Δ11350-96) together with the wild-type strain Br48 (WT). Fungal genomic DNA was digested with *Hin*dIII, separated on a 1.2% agarose gel, and hybridized with a DIG-labeled probe. (c) Relationship between the genomic copy number and the rate of DNA methylation of MGY-ΔRT in the three double deletion mutants. The solid and dashed lines represent the regression lines of the wild-type and Δ11350-22 strains, respectively, shown in Fig. 1a.

Supplementary Figure 3. Transposition-deficient MGY-ΔRT is targeted for DNA methylation at levels comparable to the wild-type MAGGY element

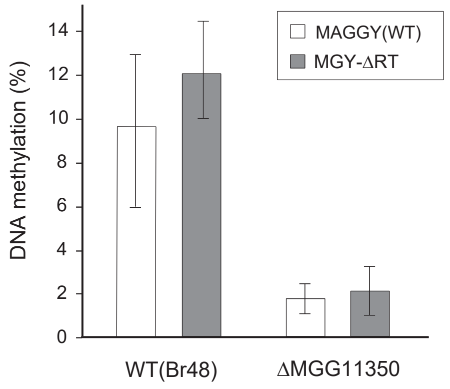

The wild-type MAGGY and its transposition-deficient mutant, MGY-ΔRT were introduced into the *P. oryzae* Br48 (wild-type) and Δ11350-22 strains, respectively. At least three independent transformants with the wild-type MAGGY or MGY-ΔRT were picked up, and subjected to DNA methylation assay by the qMSRE method. In case of MGY-ΔRT, transformants possessing 8 to 10 integrated copies of the element were selected and used in the analysis. Each bar represents the average of eight biological replicates (±SE).

Supplementary Figure 4. Construction of a single deletion mutant of Ddnm1 or Rhm51

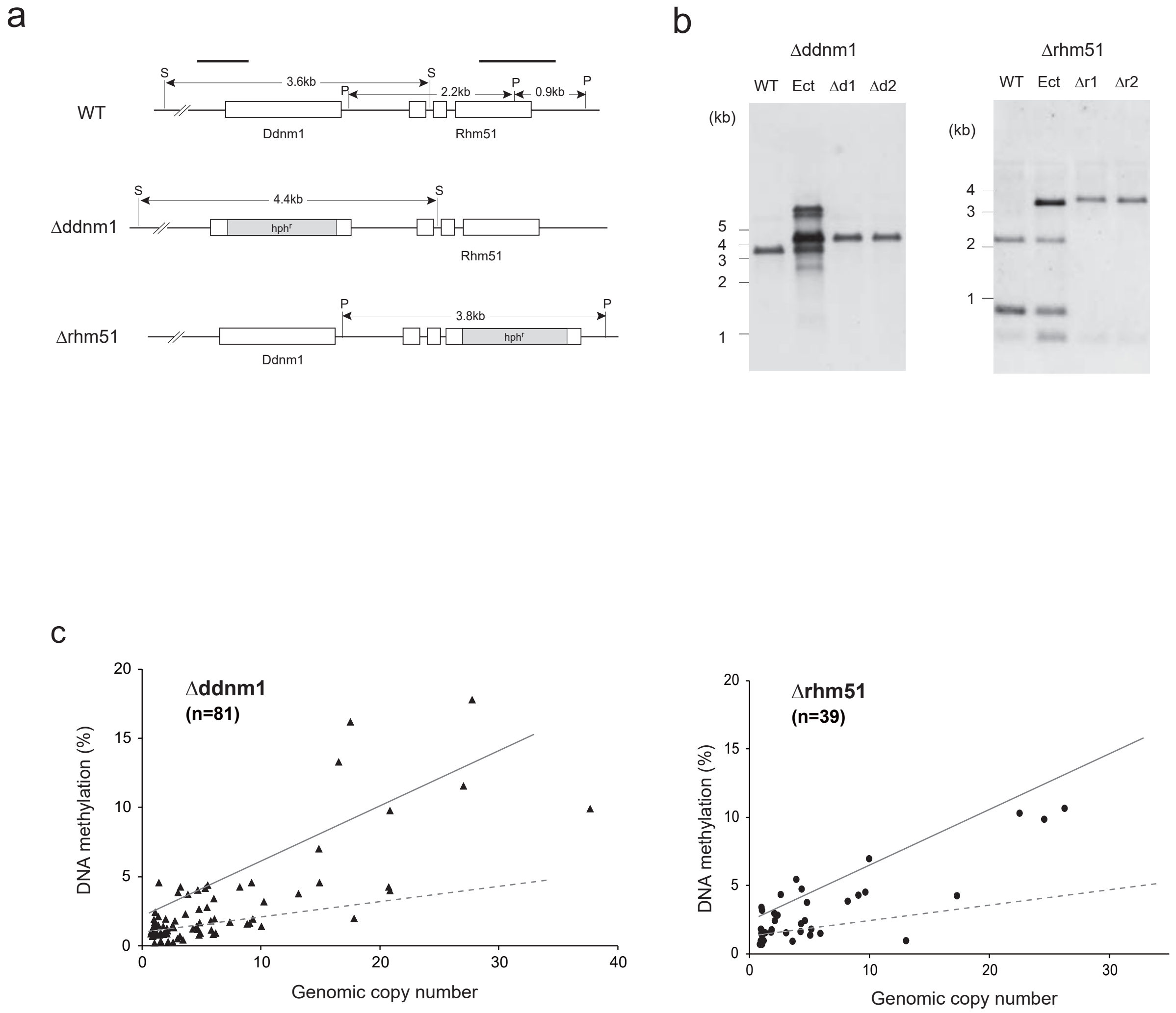

(a) Presumed genome structures of the *Rhm51* and *MGG_15577* loci in the wild type Br48 (WT), Δddnm1, and Δrhm51 strains. The deletion strains were obtained via gene targeting by homologous recombination using the hygromycin resistance gene (Hyg^r) as a selection marker. The bold line shows the postion of probes used in the Sourhtern blot analysis. The sizes of the *Stu*I and *Pvu*II fragments are given with arrowed lines. P, *Pvu* II. S, *Stu* I. (b) Genomic Southern analysis of Δddnm1, and Δrhm51. Fungal genomic DNA was digested with *Pvu* II or *Stu* I, separated on a 1.2% agarose gel, and hybridized with a DIG-labeled probe. The wild-type strain (WT) and an ectopic mutant (Ect) were also used in the analysis. (c) Relationship between the genomic copy number and the rate of DNA methylation of MAG-ΔRT in the Δddnm1, and Δrhm51 strains. The solid and dashed lines represent the regression lines of the wild-type and Δ11350-22 strains, respectively (Fig. 1a).

Supplementary Figure 5. Ddnm1 and Rhm51 are involved in heterochromatin formation to suppress MAGGY transposition.

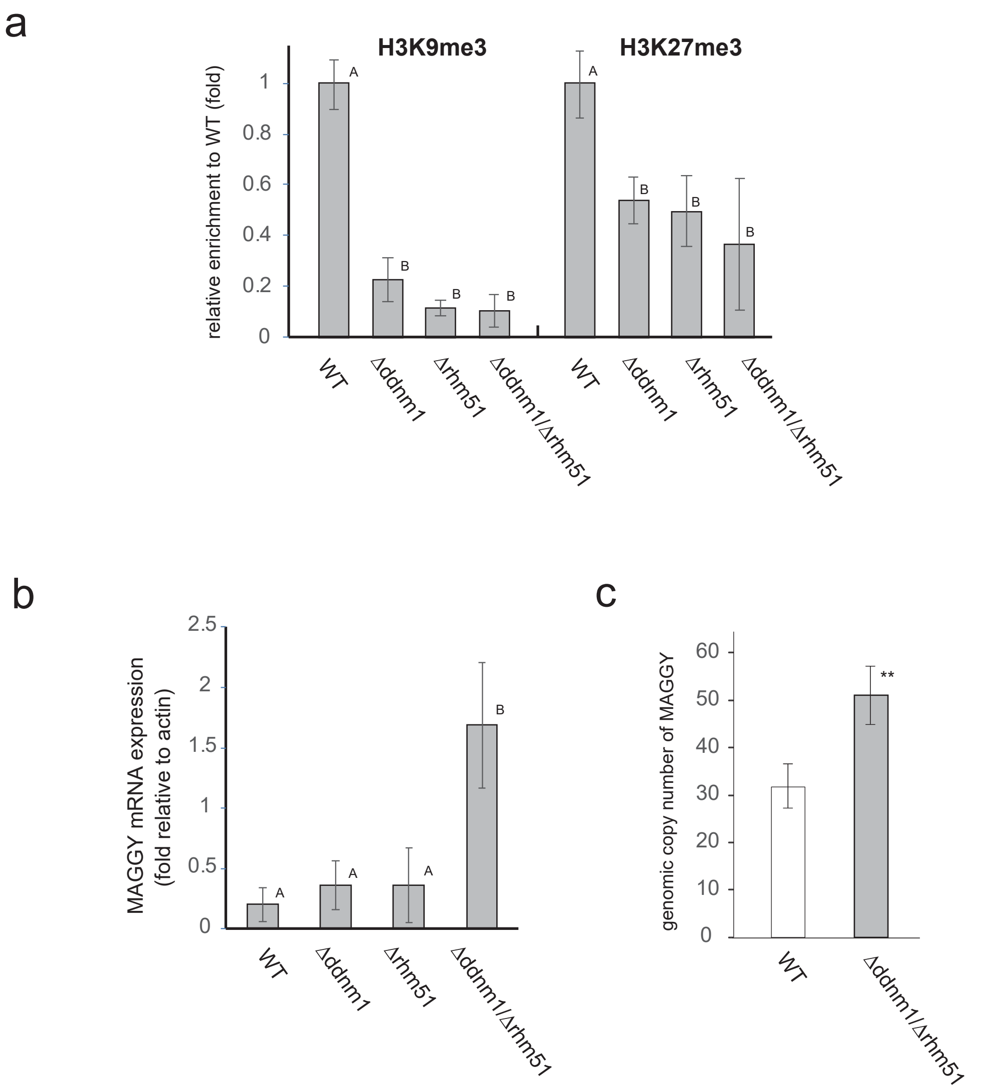

(a) ChIP–qPCR analysis of H3K9me3 and H3K27me3 at MGY-ΔRT loci in the wild-type (WT) and Δddnm1, Δrhm51 and Δddnm1/Δrhm51 cells. In the qPCR assay, a MAGGY fragment in the integrase domain was amplified using a pair of primers (5'-ctgctatccactgctcacca-3', 5'-gaagccaactttcccaatca-3'). The data was normalized to a reference actin gene. Enrichment of the histone modifications relative to WT is presented. Each bar represents the average of three replicates (±SE). Different capital letters indicate significant differences between the means (Tukey/Kramer, $p < 0.05$) (b) qRT-PCR analysis of MAGGY mRNA with the primers used in (a). The data was normalized to actin mRNA. Each bar represents the average of three replicates (±SE). Different capital letters indicate significant differences between the means (Tukey/Kramer, $p < 0.05$) (c) Intron-excision assay for assessing the transposition activity of MAGGY in the WT and Δddnm1/Δ rhm51 strains. A relative ratio of intron-less or intron-containing MAGGY DNA was measured by qPCR using sets of primers specific to “exon” junction or intron internal sequences. Each bar represents the average of five replicates (±SE). Asterisks indicate a significant difference from WT (two-tailed t-test; **, $P<0.01$).

Supplementary Figure 6. The full western blot images of Figure 2b.

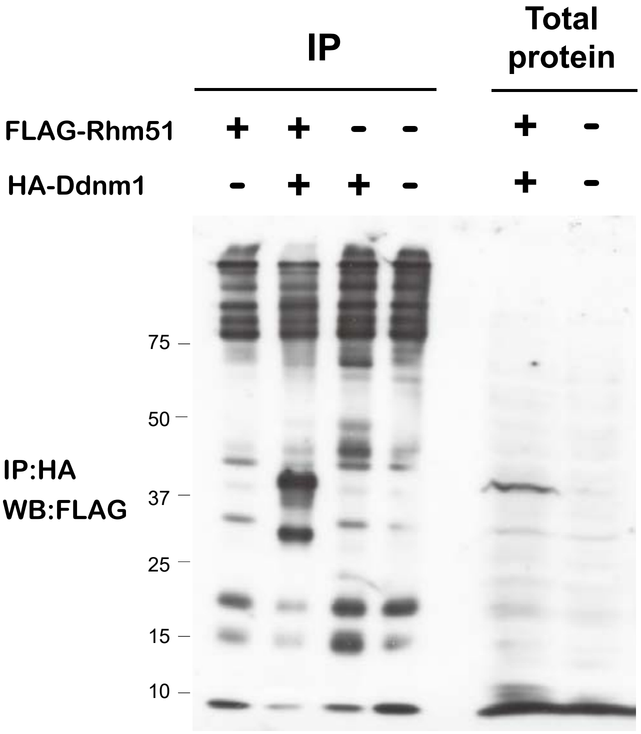

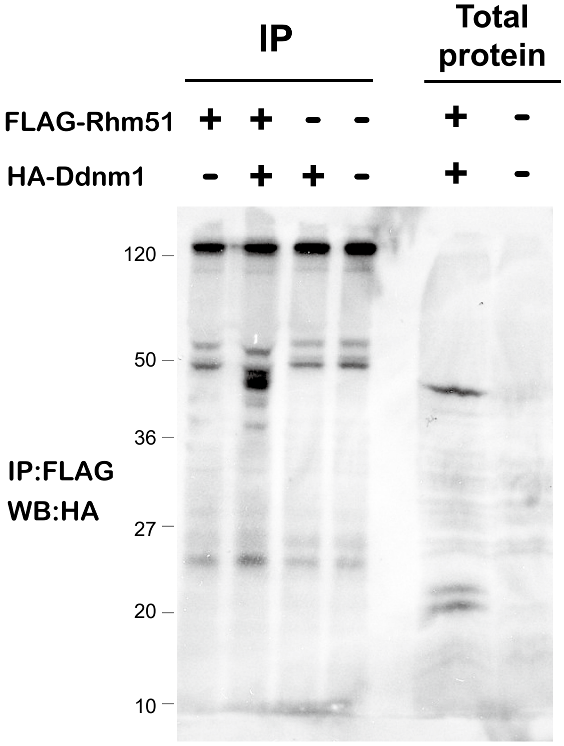

Coimmunoprecipitation assay of Ddnm1 and Rhm51. Ddnm1 and Rhm1 were tagged with HA and FLAG at the N-terminus, respectively, and introduced into *P. oryzae* cells by transformation. Cell lysates were immunoprecipitated (IP) with anti-HA or anti-FLAG antibodies, followed by western blotting (WB) with an anti-FLAG or anti-HA antibody.

Supplementary Figure 7. The Ddnm1/Rhm51 complex is involved in *de novo* DNA methylation of MAGGY but not its maintenance

a

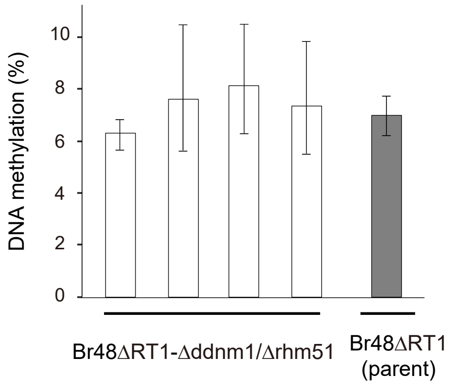

b

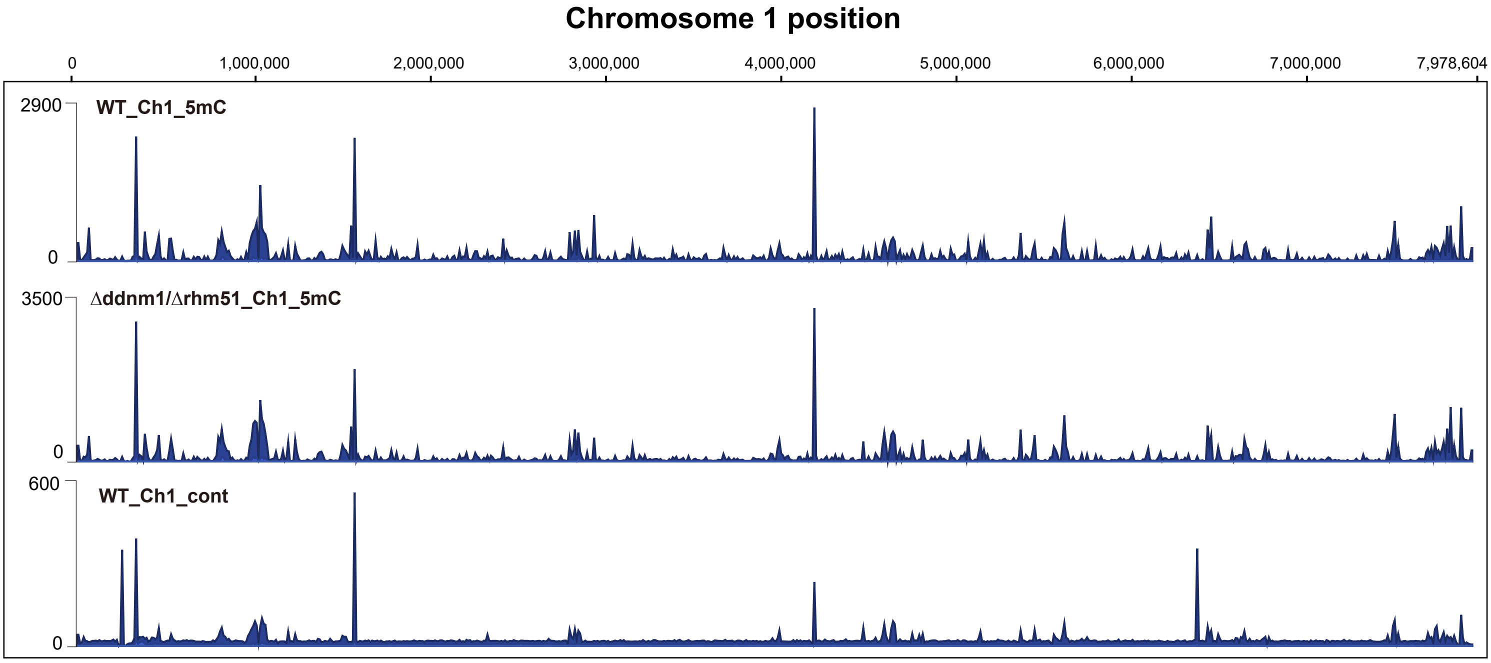

(a) Four independent Δddnm1/Δrhm51 double knock-out mutants were constructed using the parent strain Br48ΔRT that possesses 10 integrated pMGY-ΔRT copies in the wild-type background. The degrees of DNA methylation of MAGGY sequences were assessed by the qMSRE method. ach bar represents the average of three technical replicates (±SE). (b) MeDIP-seq reads obtained from the wild-type and Δddnm1/Δrhm51 strains were mapped to the genome of the *P. oryzae* reference strain (70-15). As a control, genomic DNA reads of the wild-type strain without MeDIP was used. Three million reads were sampled from each data and used in the analysis. The resulting reads coverages on the entire chromosome 1 are presented.

Supplementary Figure 8. Construction of deletion mutants of *MoRad55* and *MoRad57*

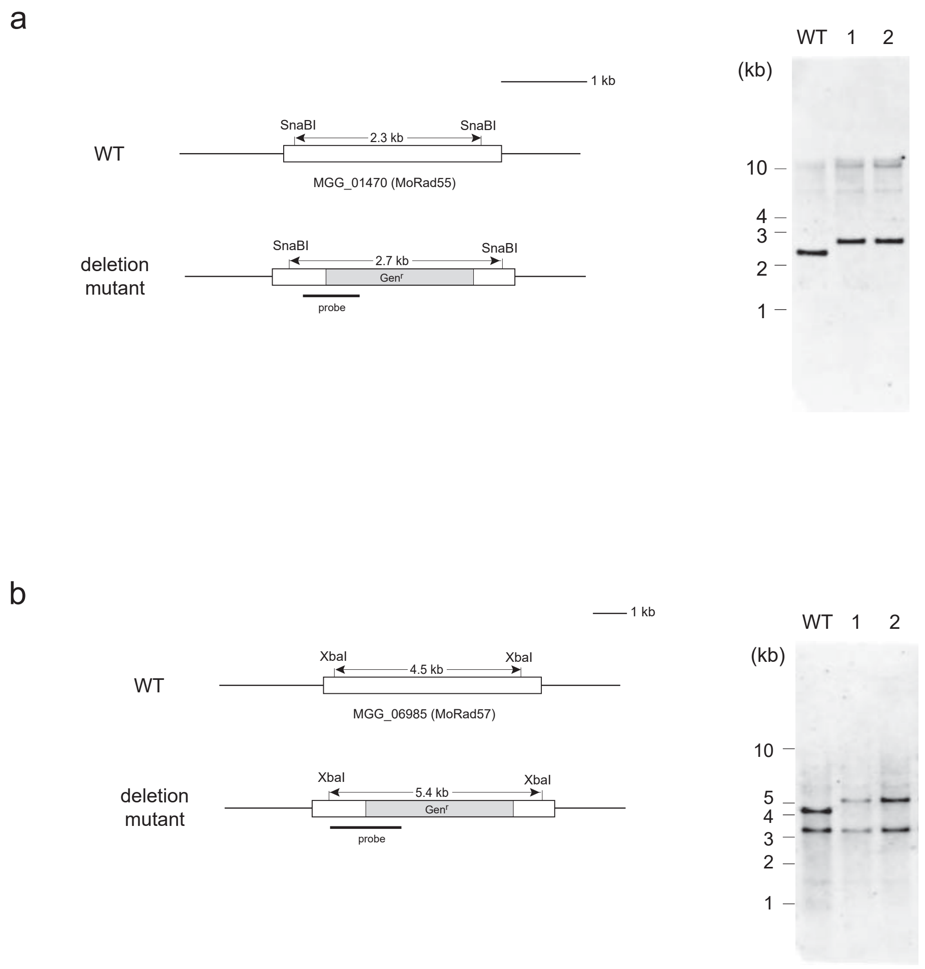

(a) Schematic representation of the MoRad55 locus (MGG_01470) in the wild-type and deletion strains, and genomic Southern blots of these strains. The open box indicates ORF. The bold line shows the postion of the probe used in the Sourhtern blot analysis. The sizes of the *Sna*BI fragments are given with arrowed lines. WT, wild-type; 1, Δmorad55; 2, Δmorad55/Δddnm1. (b) Schematic representation of the MoRad57 locus (MGG_06985) in the wild-type and deletion strains, and genomic Southern blots of these strains. The open box indicates ORF. The bold line shows the postion of the probe used in the Sourhtern blot analysis. The sizes of the *Xba*I fragments are given with arrowed lines. WT, wild-type; 1, Δmorad57; 2, Δmorad57/Δddnm1.

Supplementary Figure 9. Gene complementation of MoDCL1 and MoAGO2

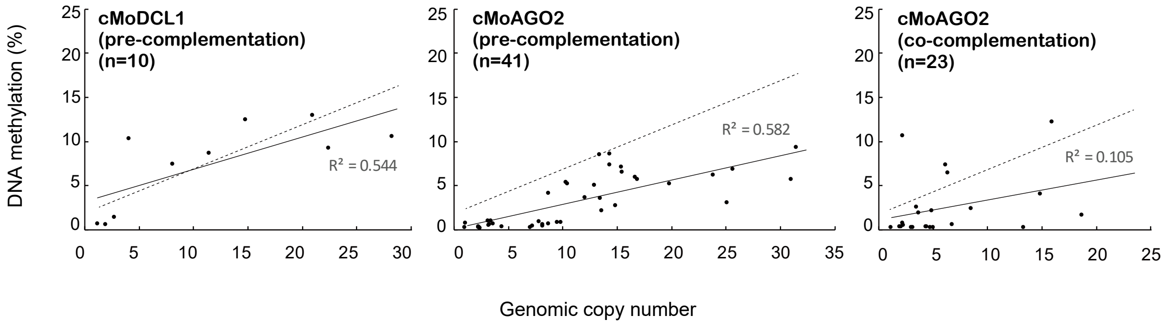

Relation between the degree of DNA methylation and genomic copy number of MGY-ΔRT in gene complementation strains of *MoDCL1* (cMoDCL1) and *MoAGO2* (cMoAGO2). In pre-complementation experiments, a genomic clone of MoDCL1 or MoAGO2 locus was first introduced into a corresponding deletion strain. Then, pMGY-ΔRT was further introduced into the resulting gene complemented strain. In co-complementation experiments, a genomic clone of *MoAGO2* and pMGY-ΔRT were simultaneously introduced into Δmoago2 strain. The dashed line represents the regression line of the wild-type strain shown in Fig. 1a.

Supplementary Figure 10. The full western blot images of Figure 5b.

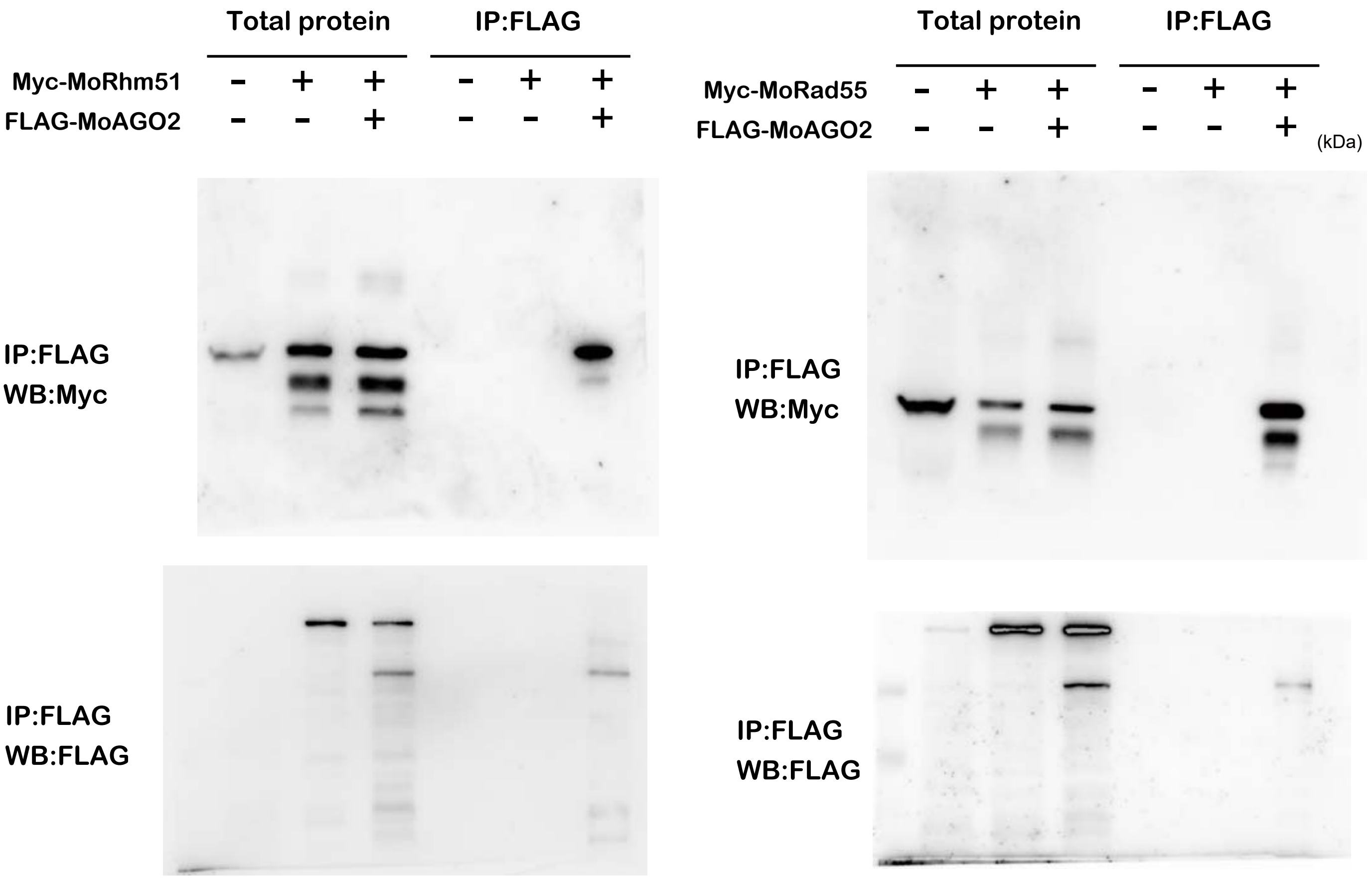

Coimmunoprecipitation assay of MoAGO2 and MoRhm51 or MoRad55. FLAG-tagged MoAGO2 and Myc-tagged MoRhm51 or Myc-tagged MoRad55 were introduced into *P. oryzae* cells by transformation. Cell lysates were immunoprecipitated (IP) with anti-Myc or anti-FLAG antibodies, followed by western blotting (WB) with an anti-FLAG or anti-Myc antibody.

Reference

37. Urashima *et al.* Molecular analysis of the wheat blast population in Brazil with a homologue of retrotransposon MGR583. Ann. Phytopathol. Soc. Jpn. **65**, 429-436 (1999).
