# Supplementary material for: Copy number-dependent DNA methylation of the Pyricularia oryzae MAGGY retrotransposon is triggered by DNA damage
Source: Commun Biol. 2021 Mar 19;4:351. doi: 10.1038/s42003-021-01836-5 (PMC7979813; doi:10.1038/s42003-021-01836-5)
Supplement: Supplementary file 3 — Description of Additional Supplementary Files [file 42003_2021_1836_MOESM3_ESM.pdf]

Description of Additional Supplementary Files

File name: Supplementary Data 1

Description: Primer pairs used in this study

File name: Supplementary Data 2

Description: All source data for the graphs and charts presented in the main figures
