# Supplementary material for: Copy number-dependent DNA methylation of the Pyricularia oryzae MAGGY retrotransposon is triggered by DNA damage
Source: Commun Biol. 2021 Mar 19;4:351. doi: 10.1038/s42003-021-01836-5 (PMC7979813; doi:10.1038/s42003-021-01836-5)
Supplement: Supplementary file 6 — Reporting Summary [file 42003_2021_1836_MOESM6_ESM.pdf]

nature research

Corresponding author(s): Hitoshi Nakayashiki

Last updated by author(s): Jan 30, 2021

Reporting Summary

Nature Research wishes to improve the reproducibility of the work that we publish. This form provides structure for consistency and transparency in reporting. For further information on Nature Research policies, see our Editorial Policies and the Editorial Policy Checklist.

Statistics

For all statistical analyses, confirm that the following items are present in the figure legend, table legend, main text, or Methods section.

n/a	Confirmed	
☐	☒	The exact sample size (*n*) for each experimental group/condition, given as a discrete number and unit of measurement
☐	☒	A statement on whether measurements were taken from distinct samples or whether the same sample was measured repeatedly
☐	☒	The statistical test(s) used AND whether they are one- or two-sided<br>*Only common tests should be described solely by name; describe more complex techniques in the Methods section.*
☐	☒	A description of all covariates tested
☒	☐	A description of any assumptions or corrections, such as tests of normality and adjustment for multiple comparisons
☐	☒	A full description of the statistical parameters including central tendency (e.g. means) or other basic estimates (e.g. regression coefficient) AND variation (e.g. standard deviation) or associated estimates of uncertainty (e.g. confidence intervals)
☐	☒	For null hypothesis testing, the test statistic (e.g. F, t, r) with confidence intervals, effect sizes, degrees of freedom and P value noted<br>*Give P values as exact values whenever suitable.*
☒	☐	For Bayesian analysis, information on the choice of priors and Markov chain Monte Carlo settings
☒	☐	For hierarchical and complex designs, identification of the appropriate level for tests and full reporting of outcomes
☒	☐	Estimates of effect sizes (e.g. Cohen's *d*, Pearson's *r*), indicating how they were calculated

Our web collection on statistics for biologists contains articles on many of the points above.

Software and code

Policy information about availability of computer code

Data collection	N/A
Data analysis	Genomics workbench software v11.0 (CLCbio). SPSS version 16.0

For manuscripts utilizing custom algorithms or software that are central to the research but not yet described in published literature, software must be made available to editors and reviewers. We strongly encourage code deposition in a community repository (e.g. GitHub). See the Nature Research guidelines for submitting code & software for further information.

Data

Policy information about availability of data

All manuscripts must include a data availability statement. This statement should provide the following information, where applicable:

- Accession codes, unique identifiers, or web links for publicly available datasets
- A list of figures that have associated raw data
- A description of any restrictions on data availability

The raw sequence data of MeDIP and GWBS were deposited in the DDBJ/ENA/GenBank database under BioProject ID PRJDB10949.

Field-specific reporting

Please select the one below that is the best fit for your research. If you are not sure, read the appropriate sections before making your selection.

☒ Life sciences ☐ Behavioural & social sciences ☐ Ecological, evolutionary & environmental sciences

For a reference copy of the document with all sections, see nature.com/documents/nr-reporting-summary-flat.pdf

Life sciences study design

All studies must disclose on these points even when the disclosure is negative.

Sample size	In ordinary experiments such as phenotypic analysis, we determined the sample size as we have been used, thus, empirically. For correlation analyses between DNA methylation and genomic copy number, the sample size was determined so that we can draw a conclusion by statistical analysis.
Data exclusions	No data was excluded in this study.
Replication	In this study, we generally repeated the experiments at least three times. For correlation analyses between DNA methylation and genomic copy number, we present the data as a sum of several independent experiments since we observed a similar tendency in every experiment.
Randomization	For correlation analyses between DNA methylation and genomic copy number, we basically used all P. oryzae transformants obtained. However, since transformants with low genomic copies of MAGGY were abundant while ones with a high copy number were rare, some transformants that were supposed to contain only a few copies of MAGGY at the stage of PCR screening for MAGGY transformants were not employed for further analysis in some cases. The samples used in the other analyses were chosen randomly.
Blinding	We believe that blinding was not applicable for the analyses we performed. We just picked up samples randomly.

Reporting for specific materials, systems and methods

We require information from authors about some types of materials, experimental systems and methods used in many studies. Here, indicate whether each material, system or method listed is relevant to your study. If you are not sure if a list item applies to your research, read the appropriate section before selecting a response.

Materials & experimental systems

n/a	Involved in the study	
☐	☒	Antibodies
☒	☐	Eukaryotic cell lines
☒	☐	Palaeontology and archaeology
☒	☐	Animals and other organisms
☒	☐	Human research participants
☒	☐	Clinical data
☒	☐	Dual use research of concern

Methods

n/a	Involved in the study	
☒	☐	ChIP-seq
☒	☐	Flow cytometry
☒	☐	MRI-based neuroimaging

Antibodies

Antibodies used	anti-H3K9me3 pAb Active Motif Cat#39161, anti-H3K27me3 pAb Active Motif Cat#39156, anti-DDDDK mAb Wako Cat#018-22381, anti-HA mAb Wako Cat#014-21881, anti-Myc-tag mAb-HRP-DirecT MBL Cat#M192-7, anti-DDDDK-tag mAb-HRP-DirecT MBL Cat#M185-7, goat anti-mouse IgG-AP conjugate Bio-Rad Cat#1706520, Anti-5-methylcytosine (5-mC) mAb Active motif Cat#39649
Validation	The antibodies against histone modifications were validated by western blots using P. oryzae mutants lacking a KMT gene responsible for the modification (Pham et al., 2015). The antibodies against the tags (DDDDK, Myc, HA) were validated also by western blots using P. oryzae transformants expressing a protein fused with the tag (this study). The antibody against 5-mC was validated by a consistency with MSRE analysis (this study).
